# The Impact of Sedation on Adenoma Detection Rate and Cecal Intubation Rate in Colonoscopy

**DOI:** 10.1155/2020/3089094

**Published:** 2020-12-16

**Authors:** Qiongmei Zhang, Zhiyu Dong, Yuanxi Jiang, Tingting Zhan, Junwen Wang, Shuchang Xu

**Affiliations:** Department of Gastroenterology, Tongji Hospital, Tongji University School of Medicine, Shanghai, China

## Abstract

**Purpose:**

To explore the effect of sedation on the quality of colonoscopy.

**Methods:**

The data collected from the Digestive Endoscopy Center of Shanghai Tongji Hospital from March 2012 to June 2019 were retrospectively analyzed. The rate of sedation and quality metrics of colonoscopy such as adenoma detection rate (ADR) and cecal intubation rate (CIR) were calculated. The logistic regression model was used to explore the relationship between sedation and quality metrics of colonoscopy. The interaction effects between experience of endoscopists and sedation on quality of colonoscopy was also investigated in subgroups stratified by total number of colonoscopies during career using the logistic regression model.

**Results:**

A total of 63,417 colonoscopies including 11,417 colonoscopies without sedation and 52,000 colonoscopies with sedation were enrolled in our study. The proportion of colonoscopy with sedation was 82.0%. The ADR and CIR were all significantly higher in cases with sedation compared with cases without sedation (ADR, 22.5% vs. 17.0%, *p* < 0.001; CIR, 94.7% vs. 91.2%, *p* < 0.001). Multivariate analysis showed that the sedation was an independent factor associated with adenoma detection (OR = 1.448, 95% CI: 1.372~1.529, *p* < 0.001) and cecal intubation (OR = 1.560, 95% CI: 1.446~1.683, *p* < 0.001). A total of 14 endoscopists with complete colonoscopy data in our database and corresponding 20,949 colonoscopies data were enrolled for further analysis. The logistic regression model yielded a similar result that sedation was an independent factor on adenoma detection and cecal intubation when the factor, experience of endoscopists, was also entered into the model as a confounder (adenoma detection, OR = 1.408, 95% CI: 1.333~1.487, *p* < 0.001; cecal intubation, OR = 1.601, 95% CI: 1.482-1.729, *p* < 0.001).

**Conclusion:**

Colonoscopy with sedation has a positive effect on ADR and CIR in all endoscopists with different experience of colonoscopy, which makes the quality of colonoscopy better.

## 1. Introduction

The aging problem of the global population is gradually intensifying while the incidence of colorectal cancer (CRC) is also increasing with advancing age. CRC is the third most common malignant tumor in the world and the second leading cause of cancer-related death [1, 2]. Colonoscopy can detect and remove potential precancerous lesions and prevent metachronous cancer by the surveillance of patients who already have had a colorectal neoplasm [3, 4]. Colonoscopy is considered to be an effective method to improve the early diagnosis rate of CRC and reduce mortality. However, the effectiveness of colonoscopy is entirely dependent upon the quality of colonoscopy [5, 6].

Many quality indicators of colonoscopy have been developed to reflect the quality of colonoscopy [7, 8] Cecal intubation rate is one of these quality indicators, which reflects the rate of complete colonoscopy and complete mucosal inspection [9]. Complete colonoscopy is essential to ensure a high-quality colon examination, and incomplete colonoscopy might lead to increased costs and inconvenience due to the repeated examination [10]. Additionally, a low CIR has been found to be associated with an increased risk of interval CRC [11]. The adenoma detection rate (ADR) is another established quality indicator. Several studies have shown that early detection and removal of adenomas could effectively prevent the development of CRC and increased ADR has been reported to be associated with a reduced risk of interval CRC and mortality [12].

Colonoscopy is the gold standard test for lower gastrointestinal tract and the preferred method for screening and monitoring CRC. However, colonoscopy may be considered a painful and embarrassing process, and this view may prevent patients from participating in the examination [13, 14]. Patients are often in a state of fear, anxiety, pain, and restlessness when they have traditional colonoscopy, which makes the operation more difficult. Patient's tolerance and compliance with the examination might decrease and even request to suspend inspection or refuse to review regularly [15–17]. As the development of sedation, colonoscopy with sedation has become widespread in the world and is considered a more comfortable way for patients. Some previous studies have demonstrated that sedation could relatively broaden the indication of colonoscopy, make patients accept examination easier, shorten the time of colonoscopy, and improve patient satisfaction [18–20]. In addition, some studies have shown that sedation can improve the quality of colonoscopy and increase the clinical effectiveness of colonoscopy [21, 22]. However, there is still much controversy surrounding the effect of sedation on quality of colonoscopy such as ADR and CIR. The interaction effects between sedation and experience of endoscopists on quality of colonoscopy still remains uncertain.

The aim of this study is to explore the role of sedation on quality of colonoscopy and provide more theoretical evidences for clinical use of sedation. The logistic regression model was used to explore the relationship between sedation and quality of colonoscopy when adjusting for other potential confounders. The role of sedation on quality of colonoscopy in subgroups stratified by experience of endoscopists was also investigated to verify the interaction effects between these two factors.

## 2. Methods

Data were collected from the endoscopic procedure database at Tongji Hospital in Shanghai, China, from 2012 to 2019. All basic information and medical records of patients including patient age and gender, the use of sedation, bowel preparation quality, endoscopic manifestation, and histological information and basic information of endoscopists who performed the colonoscopy including endoscopist age and gender and experience of colonoscopy were included in this study.

The ADR and CIR were considered primary outcome measures in our study. Adenoma detection rate was the fraction of patients undergoing screening colonoscopy who had at least one adenoma detected. Cecal intubation rate was defined as the proportion of colonoscopies in which the cecum or terminal ileum was reached.

In colonoscopy with sedation, patients received anesthesia with intravenous propofol to achieve deep sedation or general anesthesia. At the beginning of the procedure, propofol was induced at 80-120 *μ*g/kg of body weight. The maintenance dose of 20-50 *μ*g/kg was repeated depending on the response of the patient, the experience of the operator, and the technical difficulties encountered, while patients undergoing traditional colonoscopy remained awake during the procedure. All patients were monitored with pulse oximetry, continuous ECG, and noninvasive blood pressure assessed every 5 min. Sedated patients were offered supplementary oxygen with nasal catheter.

### 2.1. Statistical Analysis

The measurement data was expressed by mean and standard deviation while the count data was expressed by the number of cases and the percentage. The Student *t*-test and chi-squared test were used to compare the patient feature and outcome measures between colonoscopy with and without sedation. The logistic regression model was used to explore the relationship between sedation and both of outcome measures. Patient factors including patient age, gender, and bowel preparation and endoscopist factors including endoscopist age and gender were entered into logistic regression model as confounders.

In order to study the interaction effects between sedation and experience of endoscopists on outcome measures, we considered the total number of colonoscopies (≤500 or >500) during their career as an indicator of endoscopists' experience and enrolled the endoscopists with complete colonoscopy data during their career in the database. Endoscopists who have not been able to count the total amount of operations due to the early employment without records in our computer system and who have changed positions during the period of the study were excluded. Endoscopists that entered the endoscopy center from 2012 and did not leave until 2019 would be included in the analysis. After screening, 14 endoscopists met the inclusion criteria and were included in this study. In this part of logistic regression analysis, the total number of colonoscopies during career was also entered into the logistic regression model as a confounder. The effect of sedation on both of outcome measures was also investigated in subgroups stratified by experience of colonoscopy.

All reported *p* values were two-sided with *p* < 0.05 defined as statistically significant. All analysis were performed using SPSS 22.0 statistical software.

## 3. Results

A total of 63,417 colonoscopies were included in our study. Overall, the proportion of colonoscopy with sedation was 82.0%. 81.05% males and 82.97% females were under sedation. Among the 26 endoscopists in our endoscopy center, only 14 endoscopists had complete colonoscopy data (*n* = 20,949) during their career in our database. There were significant differences on patient age, gender, and bowel preparation quality between patients with and without sedation (patient age, 55.85 ± 13.92 vs. 55.46 ± 14.59, *p* < 0.001; male gender, 50.0% vs. 53.3%, *p* < 0.001; good bowel preparation, 96.4% vs. 97.6%, *p* < 0.001). In outcome measures, both ADR and CIR were significantly higher in patients with sedation compared with patients without sedation (ADR, 22.5% vs. 17.0%, *p* < 0.001; CIR, 94.7% vs. 91.2%, *p* < 0.001) (Table 1).

**Figure 1:**
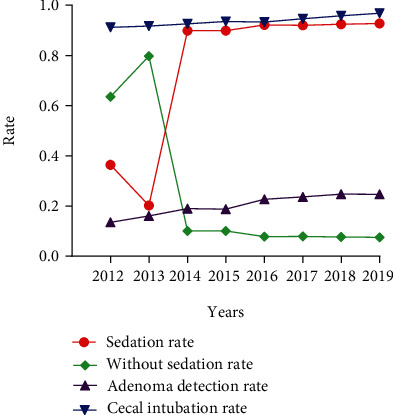
The rate of colonoscopy with or without sedation, adenoma detection rate, and cecal intubation rate in each year from 2012 to 2019.

As for annual trend in Figure 1, the proportion of patients who chose sedation increased from 36.42% in 2012 to 92.72% in 2019. The proportion of sedation increased rapidly in 2014 and maintained at a high level about 90% constantly. The ADR and CIR showed a dynamic upward trend from 2012 to 2019 as well. At the period of our study, the ADR increased from 13.53% to 24.69% while the CIR increased from 91.18% to 96.85% (Figure 1).

### 3.1. The Effect of Sedation and Interaction Effects between Sedation and Experience of Endoscopists on Adenoma Detection

In multivariate regression analysis in all colonoscopy data (model 1), the colonoscopy with sedation was an independent factor associated with higher adenoma detection rate (OR = 1.448, 95% CI: 1.372~1.529, *p* < 0.001) when controlling for other confounders (Table 2).

When the factor, total number of colonoscopies during career, was also entered into the logistic regression model (model 2), it was found that the experience of endoscopists and the patient age were significantly associated with adenoma detection in multivariate regression analysis. The colonoscopy with sedation was also an independent predictor for adenoma detection (OR = 1.408, 95% CI: 1.333~1.487, *p* < 0.001) when adjusting for these confounding factors (Table 2).

In subgroups stratified by experience of endoscopists, multivariate regression analysis showed that colonoscopy with sedation was significantly associated with adenoma detection in data of colonoscopy performed by both inexperienced endoscopists (total number of colonoscopies ≤ 500, OR = 1.339, 95% CI: 1.097~1.633, *p* = 0.004) and experienced endoscopists (total number of colonoscopies > 500, OR = 1.431, 95% CI: 1.227~1.670, *p* < 0.001) when adjusting for other confounders(Table 3).

### 3.2. The Effect of Sedation and Interaction Effects between Sedation and Experience of Endoscopists on Cecal Intubation

In multivariate analysis in all colonoscopy data (model 1), the patient gender, bowel preparation, and endoscopist gender were significantly associated with cecal intubation. When controlling for these confounders, the colonoscopy with sedation was an independent factor associated with higher cecal intubation rate (OR = 1.560, 95% CI: 1.446-1.683, *p* < 0.001) (Table 4).

When the total number of colonoscopies during career was entered into the logistic regression model (model 2), it was found that the patient gender, bowel preparation, and the endoscopist gender were significantly associated with cecal intubation in multivariate regression analysis. The colonoscopy with sedation was also an independent predictor for higher cecal intubation rate (OR = 1.601, 95% CI: 1.482-1.729, *p* < 0.001) when adjusting for these confounding factors (Table 4).

In subgroup analysis, the colonoscopy with sedation was significantly associated with cecal intubation in data of colonoscopy performed by both inexperience endoscopists (total number of colonoscopies ≤ 500, OR = 2.304, 95% CI: 1.683-3.153, *p* < 0.001) and experienced endoscopists (total number of colonoscopies > 500, OR = 1.374, 95% CI: 1.024-1.844, *p* = 0.034) when adjusting for other confounders (Table 5).

## 4. Discussion

Colonoscopy with sedation has been carried out for decades and was widely used in clinical practice. In our hospital, the sedation rate increased from 36.42% in 2012 to 92.72% in 2019 while the ADR and CIR increased year by year as the increasing sedation rate. However, there is still much controversy surrounding the effect of sedation on quality of colonoscopy. Some previous studies showed that patients who received sedation were more likely to have an enhanced CIR [23–26], but the other study demonstrated similar rates of cecal intubation among sedated and unsedated colonoscopy [27]. In our results, the colonoscopy with sedation was significantly associated with higher CIR when controlling for other patient and endoscopist factors, which confirms positive results from previous studies. As for another indicator, most of previous studies showed that the colonoscopy with sedation had no significant impact on ADR [24, 25, 28]. Researchers compared the ADR between patients with moderate or deep sedation and found that there were no significant differences on ADR between the two groups [28–30]. In contrast to previous studies, the colonoscopy with sedation was found to be associated with higher ADR as well when adjusting for other confounders in our study. For sensitive analysis, we also included all endoscopists with complete colonoscopy data during their career in our database and entered the total number of procedures during career, an indicator to reflect the experience of endoscopists, into the multivariate regression model as confounders. In sensitive analysis, the similar results about positive effects of sedation on ADR and CIR were obtained. Compared with previous researches, our study used a larger sample size and considered more factors such as endoscopist age, gender, and experience of endoscopists as confounders to adjust the effect of sedation on colonoscopy quality, which makes our results more comprehensive and reliable. In addition, some previous studies showed a higher polyp detection rate in colonoscopies with sedation [23, 26] and polyp detection rate has been considered a well-defined surrogate for ADR in recent guidelines [8], which provides indirect evidence for our results.

Based on clinical experience, we believed that experienced endoscopists might have higher capacity and perform more quality colonoscopy. Some previous studies have confirmed that more colonoscopy procedures endoscopists performed led to higher ADR and CIR [26, 31]. In order to explore whether the experience of endoscopists affected the role of sedation on colonoscopy quality and whether all endoscopists with different experiences benefited from sedation, we classified all colonoscopy data into two subgroups according to total number of colonoscopies during career and investigated the interaction effects between experience of endoscopists and sedation in subdata. In our results, the multivariate analysis showed that the colonoscopy with sedation was an independent predictor for higher ADR and CIR in both subgroups, which was consistent with our result of the multivariate analysis in model 1 and model 2. These positive results indicate that both inexperienced and senior endoscopists can benefit from sedation and perform more quality colonoscopy in colonoscopies with sedation.

The major limitation of our study is the inadequate indicators to reflect the colonoscopy quality. We investigated two important indicators for colonoscopy quality including ADR and CIR, but we neglected other indicators such as comfort and patient satisfaction. There was a lack of data on patient satisfaction due to the retrospective study, so relevant indicators could not be investigated. Furthermore, sedation might lead to more complications and financial burden for patients, which were not assessed in this study. Further research should be conducted on complications and the potential economic impact of sedation cost on colonoscopy.

## 5. Conclusion

This study showed a higher ADR and CIR in colonoscopy with sedation. The sedation was an independent predictor for higher ADR and CIR when controlling other confounders and both of inexperienced and senior endoscopists could benefit from sedation and perform more quality procedures in colonoscopies with sedation.

## Figures and Tables

**Table 1:** Patients' features with or without sedation (*n* = 63,417).

Characteristics	Colonoscopy		*p* value
	With sedation (*n* = 52,000)	Without sedation (*n* = 11,417)	
Patient age			<0.001
Mean (SD)	55.85 ± 13.92	55.46 ± 14.59	
Patient gender			<0.001
Male	26,023 (50.0%)	6,084 (53.3%)	
Female	25,977 (50.0%)	5,333 (46.7%)	
Bowel preparation			<0.001
Good	50,151 (96.4%)	11,138 (97.6%)	
Not good	1,849 (3.6%)	279 (2.4%)	
Adenoma detection rate	11,687 (22.5%)	1,940 (17.0%)	<0.001
Cecal intubation rate	49,239 (94.7%)	10,415 (91.2%)	<0.001

SD: standard deviation.

**Table 2:** Logistic regression analysis of risk factors for adenoma detection rate.

Variable	Model 1 (*n* = 63,417)		Model 2 (*n* = 20,949)	
	OR (95% CI)	*p* value	OR (95% CI)	*p* value
Patient factor				
Sedation				
Without sedation	Reference		Reference	
With sedation	1.448 (1.372-1.529)	<0.001	1.408 (1.333-1.487)	<0.001
Bowel preparation				
Good	Reference		Reference	
Not good	0.752 (0.694-0.815)	<0.001	0.761 (0.702-0.825)	<0.001
Gender				
Male	Reference		Reference	
Female	0.523 (0.503-0.544)	<0.001	0.523 (0.503-0.544)	<0.001
Age				
<50	Reference		Reference	
50-59	2.729 (2.570-2.899)	<0.001	2.727 (2.568-2.897)	<0.001
60-69	3.683 (3.482-3.895)	<0.001	3.673 (3.473-3.885)	<0.001
≥70	4.396 (4.120-4.691)	<0.001	4.401 (4.125-4.696)	<0.001
Endoscopist factor				
Gender				
Male	Reference		Reference	
Female	0.987 (0.947-1.029)	0.552	0.996 (0.955-1.038)	0.833
Age				
<40	Reference		Reference	
≥40	0.942 (0.905-0.980)	0.003	0.911 (0.875-0.949)	<0.001
Total number of colonoscopies				
≤500	—	—	Reference	
>500	—	—	1.225 (1.162-1.291)	<0.001

Model 1, all endoscopists with their colonoscopy data were enrolled; model 2, 14 endoscopists with complete colonoscopy data during career in database were enrolled.

**Table 3:** Logistic regression analysis of risk factors for adenoma detection rate in subgroups stratified by experience of colonoscopy.

Variable	≤500		>500	
	OR (95% CI)	*p* value	OR (95% CI)	*p* value
Patient factor				
Sedation				
Without sedation	Reference		Reference	
With sedation	1.339 (1.097-1.633)	0.004	1.431 (1.227-1.670)	<0.001
Bowel preparation				
Good	Reference		Reference	
Not good	0.878 (0.728-1.058)	0.171	0.792 (0.680-0.921)	0.003
Patient factor				
Gender				
Male	Reference		Reference	
Female	0.570 (0.504-0.644)	<0.001	0.527 (0.485-0.571)	<0.001
Age				
<50	Reference		Reference	
50-59	2.603 (2.151-3.149)	<0.001	2.988 (2.642-3.378)	<0.001
60-69	3.604 (3.034-4.282)	<0.001	4.078 (3.645-4.562)	<0.001
≥70	4.408 (3.603-5.393)	<0.001	4.920 (4.295-5.636)	<0.001
Endoscopist factor				
Gender				
Male	Reference		Reference	
Female	0.931 (0.783-1.107)	0.421	1.139 (1.045-1.242)	0.003
Age				
<40	Reference		Reference	
≥40	1.016 (0.812-1.271)	0.889	0.939 (0.821-1.073)	0.354

Subgroups were grouped according to the total number of endoscopists' colonoscopies (≤500 or >500) during their career.

**Table 4:** Logistic regression analysis of risk factors for cecal intubation rate.

Variable	Model 1 (*n* = 63,417)		Model 2 (*n* = 20,949)	
	OR (95% CI)	*p* value	OR (95% CI)	*p* value
Patient factor				
Sedation				
Without sedation	Reference		Reference	
With sedation	1.560 (1.446-1.683)	<0.001	1.601 (1.482-1.729)	<0.001
Bowel preparation				
Good	Reference		Reference	
Not good	1.170 (1.016-1.346)	0.029	1.161 (1.008-1.336)	0.038
Patient factor				
Gender				
Male	Reference		Reference	
Female	1.452 (1.358-1.553)	<0.001	1.451 (1.357-1.551)	<0.001
Age				
<50	Reference		Reference	
50-59	0.528 (0.474-0.587)	<0.001	0.528 (0.475-0.588)	<0.001
60-69	0.434 (0.393-0.479)	<0.001	0.436 (0.394-0.481)	<0.001
≥70	0.224 (0.202-0.249)	<0.001	0.224 (0.202-0.249)	<0.001
Endoscopist factor				
Gender				
Male	Reference		Reference	
Female	1.285 (1.192-1.385)	<0.001	1.271 (1.179-1.371)	<0.001
Age				
<40	Reference		Reference	
≥40	0.549 (0.509-0.592)	<0.001	0.565 (0.523-0.611)	<0.001
Total number of colonoscopies				
≤500	—	—	Reference	
>500	—	—	0.839 (0.763-0.921)	<0.001

Model 1, all endoscopists with their colonoscopy data were enrolled; model 2, 14 endoscopists with complete colonoscopy data during career in database were enrolled.

**Table 5:** Logistic regression analysis of risk factors for cecal intubation rate in subgroups stratified by experience of colonoscopy.

Variable	≤500		>500	
	OR (95% CI)	*p* value	OR (95% CI)	*p* value
Patient factor				
Sedation				
Without sedation	Reference		Reference	
With sedation	2.304 (1.683-3.153)	<0.001	1.374 (1.024-1.844)	0.034
Bowel preparation				
Good	Reference		Reference	
Not good	1.877 (1.145-3.077)	0.012	1.645 (1.122-2.413)	0.011
Patient factor				
Gender				
Male	Reference		Reference	
Female	1.201 (0.929-1.553)	0.161	1.371 (1.137-1.655)	0.001
Age				
<50	Reference		Reference	
50-59	0.605 (0.394-0.928)	0.021	0.525 (0.380-0.726)	<0.001
60-69	0.456 (0.314-0.663)	<0.001	0.354 (0.266-0.469)	<0.001
≥70	0.211 (0.143-0.311)	<0.001	0.157 (0.117-0.210)	<0.001
Endoscopist factor				
Gender				
Male	Reference		Reference	
Female	1.432 (0.958-2.139)	0.080	1.132 (0.931-1.376)	0.214
Age				
<40	Reference		Reference	
≥40	0.472 (0.297-0.750)	0.001	1.709 (1.174-2.487)	0.005

Subgroups were grouped according to the total number of endoscopists' colonoscopies (≤500 or >500) during their career.

## Data Availability

The colonoscopy data used to support the findings of this study are available from the corresponding author upon request.
